# Editorial: Advances in brain imaging and stimulation methods for cognitive function investigation

**DOI:** 10.3389/fnhum.2023.1280782

**Published:** 2023-08-30

**Authors:** Ulrich Palm, Giovanni Assenza, Charlotte A. Boettiger, Zhen Yuan

**Affiliations:** ^1^Department of Psychiatry and Psychotherapy, Klinikum der Universität München, Munich, Germany; ^2^Medical Park Chiemseeblick, Bernau, Germany; ^3^Campus Bio-Medico, University of Rome, Rome, Italy; ^4^Biomedical Imaging Research Center, Bowles Center for Alcohol Studies, University of North Carolina at Chapel Hill, Chapel Hill, NC, United States; ^5^Centre for Cognitive and Brain Sciences, University of Macau, Macau, China

**Keywords:** brain stimulation, brain imaging, clinical application, neuropsychology, transcranial magnetic stimulation

The Research Topic “Advances in brain imaging and stimulation methods for cognitive function investigation” gathers basic research and clinical results of experimental brain imaging and stimulation techniques which are used to investigate mechanisms of cognitive function and dysfunction.

The broad range of variety of possible preclinical and clinical application of brain stimulation methods and brain imaging is reflected by two main directions in this Research Topic: (1) the use of brain stimulation methods in a clinical or neuropsychological perspective and (2) the use of neurobiological markers to detect neurophysiological changes by brain stimulation. Both groups contain three articles each.

Clinical use of brain stimulation methods is shown in a case report by Fickling et al. investigating the effects of translingual neurostimulation (TLNS) and physical rehabilitation to facilitate recovery from traumatic brain injury in a former soldier. The effect of rehabilitation was larger when applying physical therapy plus brain stimulation than physical therapy alone. Clinical and cognitive improvement was corresponding to an increase in event-related potentials of P300 and N400 response amplitudes.

Holczer et al. conducted a review on the use of transcranial magnetic stimulation and transcranial direct current stimulation in patients with Alzheimer's disease and mild cognitive impairment. They included 23 studies in their review and found a high risk of bias due to heterogeneity of studies. Overall, data were not robust enough for any recommendation, however, transcranial magnetic stimulation seemed to have a potential effect.

Finally, Majdi et al. discuss in their hypothesis paper that brain stimulation effects potentially may not be elicited by the stimulation of brain tissue itself, but rather by co-activation of cranial and cervical nerves. Therefore, the authors analyzed six studies reporting on brain stimulation and neuropsychological or neuroimaging results and found that central effects may also be provoked by peripheral neurotransmitter release. They call for a better distinction between transcranial and transcutaneous effects in future brain stimulation studies.

In the group of articles dealing with neuropsychological and neuroimaging effects of brain stimulation methods, Pihlaja et al. report on a study involving 25 healthy subjects undergoing transcutaneous vagus nerve stimulation and a go/nogo-task. Event-related potentials P3 and N2 were recorded and showed no correlation between vagus nerve stimulation and brain activation. Authors suggest that vagus nerve stimulation leads to a lower need for brain activation during a cognitive task, however data has to be interpreted cautiously.

Wang et al. present a plot of a brain imaging study including three persons undergoing weekly sessions of functional connectivity magnetic resonance imaging during 1 year. Aim of this study is to gain better insight in stability or variability of brain networks and influencing factors over a long time.

Finally, Riddle et al. present a guide for the concurrent application of transcranial magnetic stimulation and functional connectivity magnetic resonance imaging. The authors discuss the crucial involvement of brain stimulation parameters, e.g., strength, duration, repetition, on imaging results and suggest solutions for induced artifacts in experimental design and post-processing.

## Author contributions

UP: Writing—original draft. GA: Writing—review and editing. CB: Writing—review and editing. ZY: Writing—review and editing.

